# Mitochondrial Signaling: Nitric Oxide Synthesis by Cytochrome c Oxidase and Its Oxygen Sensitivity Are Modulated by Adenine Nucleotides

**DOI:** 10.64898/2026.08.09.743791

**Published:** 2026-08-10

**Authors:** Pablo R. Castello, Kerri A. Ball, Robert O. Poyton

## Abstract

Nitrite can be reduced to nitric oxide (NO) by several heme- and molybdenum-containing proteins, including mitochondrial cytochrome c oxidase (Cco). This activity, designated Cco/NO, has been implicated in hypoxic signaling, but its regulation and quantitative significance relative to other NO-producing systems remain uncertain. We examined its modulation by adenine nucleotides using detergent-solubilized yeast and mouse brain mitochondria supplied with 1 mM nitrite and an ascorbate/TMPD/cytochrome c electron-donor system. ADP and ATP differentially modulated Cco/NO activity, and ADP extended measurable NO formation across the entire oxygen range tested, up to the assay ceiling of 175 µM O₂. Nucleotide regulation was also isoform-dependent: ATP slightly inhibited Va-containing Cco but strongly stimulated Vb-containing Cco under anoxic conditions. Rates normalized to cytochrome aa₃ demonstrate multi-turnover nitrite-reductase capacity under these substrate-driven assay conditions. Both the cellular ADP/ATP ratio and subsequently assayed Cco/NO activity increased transiently following a hypoxic shift. These findings establish metabolic and isoform-dependent gating of the catalytic capacity of Cco/NO; they do not establish its fractional contribution to total cellular NO or its operation at physiological nitrite concentrations in intact, coupled mitochondria.

This research was supported by CONICET Grant PIP 706 (research team member P.R.C.) and National Institutes of Health Grant GM30228 to R.O.P.

## Full Text Availability

The license terms selected by the author(s) for this preprint version do not permit archiving in PMC. The full text is available from the preprint server.

